# Vaccination in the Era of Immunosuppression

**DOI:** 10.3390/vaccines11091446

**Published:** 2023-09-01

**Authors:** Fatima Alnaimat, Jaleel Jerry G. Sweis, Jacqueline Jansz, Zeel Modi, Supritha Prasad, Ayman AbuHelal, Christen Vagts, Hali A. Hanson, Christian Ascoli, Richard M. Novak, Ilias C. Papanikolaou, Israel Rubinstein, Nadera Sweiss

**Affiliations:** 1 Department of Internal Medicine, Division of Rheumatology, School of Medicine, University of Jordan, Amman 11942, Jordan; 2 Royal Jordanian Medical Services, Amman 11855, Jordan; jerrysweis@gmail.com; 3 Department of Medicine, University of Illinois Chicago, Chicago, IL 60612, USA; jjansz2@uic.edu (J.J.); modizeel@gmail.com (Z.M.); spprasa2@uic.edu (S.P.); 4 Jordan University Hospital, Amman 11942, Jordan; ayman.helal@hotmail.com; 5 Department of Medicine, Division of Pulmonary Critical Care Sleep and Allergy, University of Illinois Chicago, Chicago, IL 60612, USA; cvagts2@uic.edu (C.V.); cascoli@uic.edu (C.A.); irubinst@uic.edu (I.R.); 6 College of Pharmacy, University of Illinois Hospital & Health Sciences System, Chicago, IL 60612, USA; hramir7@uic.edu; 7 Division of Infectious Diseases, University of Illinois, Chicago, IL 60612, USA; rmnovak@uic.edu; 8 Department of Respiratory Medicine, Sarcoidosis Clinic, Corfu General Hospital, 49100 Corfu, Greece; icpapanikolaou@hotmail.com; 9 Division of Rheumatology, Department of Medicine, University of Illinois Chicago, Chicago, IL 60612, USA; nsweiss@uic.edu

**Keywords:** vaccination, autoimmune, immunosuppressed, immune response

## Abstract

Patients with autoimmune inflammatory rheumatic diseases (AIIRDs) are at increased risk for severe infections. Vaccine responses and safety profiles may differ between AIIRD patients and the general population. While patients with autoimmune inflammatory rheumatic diseases (AIIRDs) often experience diminished humoral responses and reduced vaccine efficacy, factors such as the type of immunosuppressant medications used and the specific vaccine employed contribute to these outcomes. Notably, individuals undergoing B cell depletion therapy tend to have poor vaccine immunogenicity. However, despite these considerations, vaccine responses are generally considered clinically sufficient. Ideally, immunosuppressed AIIRD patients should receive vaccinations at least two weeks before commencing immunosuppressive treatment. However, it is common for many patients to already be on immunosuppressants during the immunization process. Vaccination rarely triggers flares in AIIRDs; if flares occur, they are typically mild. Despite the heightened infection risk, including COVID-19, among AIIRD patients with rheumatoid arthritis, systemic lupus erythematosus, sarcoidosis, and other diseases on immunosuppressants, the vaccination rates remain suboptimal. The future directions of vaccination in the era of immunosuppression will likely involve customized vaccines with enhanced adjuvants and alternative delivery methods. By addressing the unique challenges faced by immunosuppressed individuals, we may improve vaccine efficacy, reduce the risk of infections, and ultimately enhance the health outcomes. Additionally, clinical trials to evaluate the safety and efficacy of temporarily discontinuing immunosuppressants during vaccination in various AIIRDs are crucial.

## 1. Introduction

Autoimmune inflammatory rheumatic diseases (AIIRDs) represent a spectrum of distinct conditions that exhibit comparable clinical, laboratory, and immunological features. These disorders are characterized by an exaggerated immune response that targets self-antigens [1]. The development of AIIRDs is influenced by genetic predisposition [2], environmental factors, hormonal imbalances [3], and stressful life events [4,5].

AIIRDs are grouped into three categories: autoinflammatory conditions (e.g., ankylosing spondylarthritis), autoimmune disorders (e.g., systemic lupus erythematosus (SLE)), and conditions that exhibit overlapping characteristics, with rheumatoid arthritis being a notable example [1].

Due to immune system dysregulation and immunosuppressive medications, patients with autoimmune inflammatory rheumatic diseases (AIIRDs) are more susceptible to severe infections [6]. These patients experience worse outcomes from illnesses that vaccinations can prevent, such as influenza and *Streptococcus pneumoniae* pneumonia [7,8]. Immunization is a highly impactful public health intervention as vaccines are proven to save lives, prevent disabilities, lower infection risk, and, consequently, significantly reduce infection-associated mortality and morbidity [9]. Studies have shown low vaccination rates among patients with AIIRDs. Moreover, the immunogenicity and safety of various vaccines may differ in patients with AIIRDs compared to the general population. Thus, adjusted vaccine indications and treatment schedules may benefit AIIRD patients [10]. 

This review article explores the immunological mechanisms underlying vaccine response in patients with AIIRDs receiving immunosuppressive therapy, the impact of immunosuppression on vaccine immunogenicity, and the influence of immunosuppressant medications on vaccination outcomes in AIIRD patients. Additionally, we discuss the utilization and efficacy of vaccines in AIIRD patients receiving immunosuppressants and explore future directions for research and advancements in vaccination strategies for AIIRD patients in the context of immunosuppression.

## 2. Immunological Mechanisms Underlying Vaccine Response

Immunization can be delivered passively, via the transfer of preformed antibodies, or actively, via the introduction of a pathogen-derived immunogen. The latter stimulates the body to develop immunity and thus confers longer-lasting protection [11]. Vaccines are a revolutionary development in medicine. Historically, vaccines have been classified into four types: live attenuated, inactivated (killed), subunit (purified antigen), and toxoids (inactivated toxic compounds) [12]. New forms of vaccines, such as mRNA, DNA, and virus-vectored vaccines, are also in current use or in development and have been made widely available in response to the SARS-CoV-2 pandemic. Although the technology for mRNA vaccines is not new and has existed for some time, it was the first time mRNA vaccines successfully completed all clinical trial phases and were licensed for widespread use in SARS-CoV-2 vaccinations [13,14]. 

The immune system is divided into two parts: innate and adaptive. Anatomic barriers such as skin and mucous membranes, physiological barriers such as body temperature and gastric acidity, complement pathways, pattern recognition receptors, and inflammation are all part of the innate immune system. Upon vaccination, the body utilizes this innate system as an initial step to recognize the vaccine-introduced antigen as a foreign pathogen. This recognition is facilitated by engineered epitopes, which are small subregions on antigens, to aid in the antigen’s engulfment by antigen-presenting cells (APCs) that express major histocompatibility complex proteins, thereby prompting the adaptive immune system response [15]. This activation of the adaptive immune system allows it to recognize and remember the specific vaccine antigen, thereby establishing a long-lasting immune memory.

**Figure 1:**
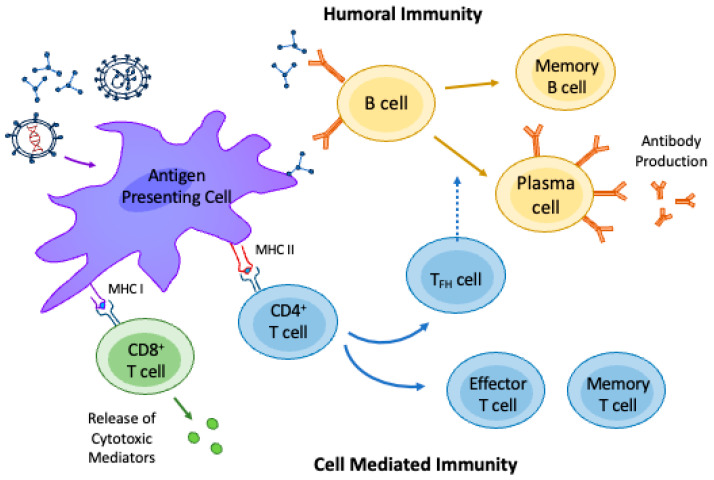
Immune response to vaccination. Once administered, the vaccine particles are phagocytosed by the innate immune system’s antigen-presenting cells (APCs). These cells process the engulfed particles and subsequently present the antigens to adaptive immune system cells. The presentation of antigens by APCs depends on the type of vaccine-incorporated components (i.e., protein subunit with or without a protein-bound adjuvant, viral DNA or mRNA vaccine, viral particles, viral or bacterial live attenuated vaccines, etc.). In general, viral antigens are presented on major histocompatibility complex type (MHC)-1 peptides to cytotoxic CD8+ T cells to activate cell-mediated immunity and viral host defense through the release of cytotoxic mediators (mainly perforin and granzyme). Bacterial and parasitic antigens are presented on MHC-2 peptides to CD4+ T cells that stimulate effector and memory T cell responses. Follicular T cells (TFH cells) aid in B cell activation and differentiation into plasma cells, which produce antigen-specific high-affinity antibodies. B cell activation may also be independent of T cells through direct interaction with the vaccine particles or through interaction with an APC. The inherent function of the APCs, T cells, and B cells involved in the immune response to vaccine may be altered by the presence of AIIRDs and the use of immunosuppressive therapy.

Viral antigens are generally bound with MHC I proteins and presented to cytotoxic CD8+ T cells. These cytotoxic T cells lead to the destruction of the pathogen, otherwise known as cell-mediated immunity. This component of the adaptive immune system recognizes pathogens only after processing and presentation via APCs and generates immunological memory [12]. In contrast to cell-mediated immunity, humoral immunity involves memory B cells or antibody-producing plasma cells. Humoral immunity is triggered when a viral, bacterial, or parasitic antigen is bound to a MHC II protein and presented to a CD4+ helper T-cell [15]. This T-helper cell stimulates B cells to mature into plasma cells and produces antigen-specific antibodies, first IgM and then IgG, conferring longitudinal immunity. B cells may also act alone, in which case antigens are recognized in their native form and induce the activation of numerous other B cells [15]. Figure 1 summarizes the immune response to vaccination.

## 3. Impaired Immune Response in AIIRD Patients

Immune dysregulation is present in individuals with AIIRDs, primarily from disease progression or secondarily through immunosuppressive treatment for disease management [16]. The most common conditions that lead to immunosuppression include malignancies, congenital or acquired immunodeficiencies, autoimmune diseases requiring treatments, and organ transplants [17]. While vaccination may rely on cellular and humoral immunity to stimulate a protective response to future antigen exposure, various inflammatory diseases are characterized by defects in key innate and adaptive immune mechanisms that make these patient populations particularly vulnerable. 

Autoantigens have been implicated in the immune dysregulation of various autoimmune diseases. In systemic lupus erythematosus (SLE), nucleic acid self-antigens derived from dying cells are presented to dysregulated T cells, leading to aberrant patterns of differentiation characterized by decreased regulatory T cell production and unchecked CD4+ T cell expansion [18]. Autoreactive B cells are stimulated by CD4+ T cells, resulting in the production of anti-nuclear antibodies that cause downstream organ dysfunction through complement activation, immune complex deposition, and altered immune effector cell function [19]. With such immune dysregulation underlying the disease process, inherent immune deficiency is associated with SLE [20].

Other notable diseases characterized by dysregulated immune response include sarcoidosis and idiopathic pulmonary fibrosis (IPF). Sarcoidosis is an immune-mediated disorder that manifests as multisystem granulomatous inflammation [21]. Chronic immune stimulation from an unknown source is a hallmark of the disease that leads to lymphocyte anergy, exhaustion, and depletion [21,22,23,24]. In IPF, repetitive pulmonary epithelial injury results in chronic inflammation characterized by profibrotic macrophages, fibrocytes, and various T cell subsets [25,26], leading to end-stage pulmonary fibrosis. Organ dysfunction and fibrosis, because of their respective immunological mechanisms, lead to diminished host defenses in both sarcoidosis and IPF [25]. 

## 4. Mechanisms of Immunosuppression and the Impact on Vaccine Immunogenicity 

AIIRD patients are at an increased risk of vaccine-preventable infections compared to the general population [27,28]. Notably, patients with AIIRDs are at increased risk of influenza, pneumococcal, herpes zoster, and human papillomavirus infections, further supporting the need for vaccination [29]. Apart from the defects in immunity conferred by the dysregulated immune mechanisms that underlie these diseases, the frequent need for immunosuppressive medications as treatment contributes to the overall vulnerability of these populations. These medications carry an increased risk for infections, and some of which are preventable with vaccination [15,30].

Non-specific immunosuppressants that suppress innate and adaptive immunity arms include glucocorticoids, methotrexate, mycophenolate, and azathioprine. Hydroxychloroquine, an antimalarial used in treating SLE, RA, and sarcoidosis, suppresses cellular immunity by diminishing the antigen-presenting ability of numerous innate immune cells [15]. Finally, monoclonal antibodies to various cytokines (e.g., tumor necrosis factor, interleukins 1 and 6) and cell receptors (e.g., CD20) may have varying suppressive effects on cell-mediated and humoral immunity based on their downstream targets [15]. 

Despite immense vaccination success, research is ongoing to understand immunosuppressive drugs’ impact on vaccination immunogenicity and responses.

Studies assessing the immunogenicity of mRNA-based SARS-CoV-2 vaccinations utilizing the anti-SARSCoV-2 spike IgG revealed that immunosuppressed patients exhibit an adequate immune response [31]. In addition, the likelihood of positive seroconversion among immunosuppressed patients with autoimmune conditions was higher than among transplant recipients [32]. Influenza vaccination immunogenicity had been evaluated in 26 studies, mostly among patients with rheumatoid arthritis, systemic lupus erythematosus, and granulomatosis with polyangiitis [33]. Similar rates of immunogenicity among patients and healthy controls were reported, except for those on rituximab, whose responses were severely impaired.

Besides the humoral response, as noted by the study above, cell-mediated immunity is also important to vaccine efficacy. Factors such as the type of immunomodulator play a vital role in T cell response. In patients with AIIRDs, the greatest decrease in SAR-CoV-2 vaccine response was observed in individuals using B cell-depleting agents, followed by TNF inhibitors [15,34]. However, despite attenuated responses due to various factors, current recommendations still call for certain vaccinations in immunosuppressed patients due to their protective benefits (see recommendations section below) [10,29]. 

## 5. Effects of Specific Immunosuppressant Medications on Vaccine Response in Patients with AIIRDs

Given the increased risk of vaccine-preventable infections, such as influenza, pneumococcal infections, herpes zoster, COVID-19, and human papillomavirus (HPV) [27,28,29], most recent guidelines expanded indications for some vaccines in patients with AIIRDs. They recommended administering vaccines in very few instances [10,16,29]. Patients with AIIRDs should ideally be vaccinated before starting immunosuppressive therapies; however, in many cases, patients take immunosuppressants at the time of vaccine administration. These medications can block the humoral response to vaccines leading to a reduction in antibodies and the overall protective effect [15]. The degree to which the immune response is blunted varies based on specific medications and vaccines. Most studies examined immunogenicity after pneumococcal, influenza, or SARS-CoV-2 vaccines. While the literature has demonstrated a diminished vaccine response in patients with AIIRDs, responses are not completely absent and are largely considered adequate [15,17]. Moreover, most recent guidelines expanded indications for some vaccines in patients with AIIRDs and recommended administering vaccines in very few instances [10,16,29].

Regarding specific medications, glucocorticoids have a dose-dependent negative effect on humoral responses, especially at doses greater than 7.5–10 mg daily [35,36]. Per ACR recommendations [10], lowering the glucocorticoid dose below 20 mg/day for most vaccinations is recommended. However, this recommendation does not apply to the influenza vaccine. The exemption is due to the seasonal nature of the virus and the requirement to administer the vaccine annually. Lowering the steroid dose annually to administer the influenza vaccine may pose challenges in effectively managing the underlying disease, and the ACR conditionally recommends “high-dose or adjuvanted influenza vaccination” for patients with AIIRDs on immunosuppression [10]. Methotrexate has also been associated with reduced humoral immune response [37,38,39], particularly with higher doses above 0.4 mg/kg/week [29]. Per the 2022 ACR guidelines, it is recommended to withhold MTX for a minimum of 2 weeks after influenza vaccination if disease activity permits. For other non-live attenuated vaccines, the ACR advises continuing MTX treatment. However, when it comes to live attenuated vaccines, the ACR guidelines suggest stopping this medication for 4 weeks before and up to 4 weeks after vaccination [10]. Sulfasalazine and hydroxychloroquine have no negative effect on vaccine response [35]. Interestingly, if SLE patients taking steroids or immunosuppressants were also on hydroxychloroquine, administering the Influenza A/H1N1 vaccine resulted in a comparable seroconversion rate to those with SLE who were not on immunosuppression [36]. 

Azathioprine in doses of more than 3.0 mg/kg/day, mycophenolate mofetil, and cyclophosphamide substantially reduce the immunogenicity of vaccines [29,35]. Little data exist on the effect of leflunomide on vaccine immunogenicity. A single study investigated leflunomide’s impact on humoral immunity after SARS-CoV-2 vaccination, employing an inactivated whole virus vaccine [40]. 

The ACR guidelines recommended maintaining azathioprine, mycophenolate mofetil, cyclophosphamide, and leflunomide when receiving all non-live attenuated vaccines [10]. However, for live attenuated vaccines, it is advised to halt azathioprine, mycophenolate mofetil, and oral cyclophosphamide for 4 weeks before up to 4 weeks after vaccination. For intravenous cyclophosphamide, it is recommended to be discontinued one dosing interval before and resumed 4 weeks after [10]. 

Tumor necrosis factor inhibitors and JAK kinases have shown mixed results between a reduced and normal vaccine response, although still protective, with vaccination [15,27,41]. Data from the NorVac study showed that AIIRD patients on TNF inhibitors in combination with methotrexate or azathioprine manifested a reduced serological response to SARS-CoV-2 vaccinations [42]. Similar findings of reduced immunogenicity to the SARS-CoV-2 vaccinations were also shown in patients with inflammatory bowel disease treated with infliximab and tofacitinib [43]. For non-live attenuated vaccines, the ACR conditionally recommends proceeding with vaccination without interruption of these treatments. However, regarding live attenuated vaccines, the ACR conditionally recommends temporarily discontinuing JAK inhibitors for 1 week before vaccination and waiting 4 weeks after vaccination to resume the treatment. Similarly, for TNF inhibitors, the ACR suggests discontinuing the therapy for one dosing interval before and resuming it 4 weeks after vaccination [10].

Abatacept (CTLA4 co-stimulation blocker) showed lower proportions of vaccine responders, whereas no detrimental effect on vaccine response was found with the use of IL-6 inhibitors, IL-12–IL-23, and IL-17 inhibitors [35]. However, for all these biologics, the ACR guidelines conditionally recommend holding the treatment for one dosing interval before and resuming it 4 weeks after vaccination [10].

Finally, rituximab, a therapy that specifically depletes CD20-positive B cells, has consistently been linked to a notable decline in vaccine response. This is likely attributed to the role of B cells in generating the humoral immune response [15,44]. B cell depletion was the primary independent factor that significantly influences the antibody response to SARS-CoV-2 vaccination in patients treated with rituximab [34]. However, patients with AIIRDs treated with rituximab were shown to generate a strong T cell response to mRNA COVID-19 vaccines despite reduced humoral responses [45].

The ACR advises administering the influenza vaccine regardless of rituximab therapy. However, for all other non-live attenuated vaccines, the timing of vaccine administration is conditionally recommended to align with the next rituximab dose and to withhold rituximab treatment for at least 2 weeks after vaccination [10]. In cases of the need to administer live attenuated vaccines to AIIRD patients on rituximab therapy, the ACR guidelines conditionally recommend discontinuing the treatment for 6 months before vaccination and resuming it 4 weeks after vaccination [10]. 

Studies have employed seroconversion rates as a useful metric for assessing vaccine efficacy, specifically by measuring the binding and neutralizing antibody titers targeting the SARS-CoV-2 spike protein post-vaccination [32]. These measures could be valuable, especially for patients on immunosuppressants known to have a diminished vaccine response, to determine vaccine effectiveness and guide decisions on potential revaccination with booster doses when necessary [42]. 

## 6. Vaccines Utilization and Efficacy in Specific AIIRD Groups on Immunosuppression

Despite the well-established knowledge that patients with AIIRD are at a higher risk of infections, including COVID-19, there remains a concerning issue of low vaccination rates within this population [7,46].

For instance, a study involving 3920 RA patients from the COMORA cohort across 17 countries demonstrated significantly low rates of influenza and pneumococcal vaccinations [47]. Similarly, a questionnaire study conducted by Haroon et al. found that only 11% of outpatient rheumatology patients received influenza and pneumococcal vaccines [48]. Moreover, a survey among patients with sarcoidosis revealed that 27% of respondents were reluctant to accept the COVID-19 vaccine due to concerns about its safety and efficacy [49]. Another notable reason for non-vaccination reported in the studies was the lack of vaccine offered to patients [48,50].

These and other studies describe significantly limited vaccination rates among patients with AIIRDs [51,52]. Both lower- and higher-income countries have reported decreased vaccination rates. Interestingly, hesitancy or avoidance of vaccination is observed not only with newly developed SARS-CoV-2 vaccines but also with long-established anti-pneumococcal and influenza vaccines. The reasons behind this phenomenon are multifaceted. One factor is the lack of strong encouragement and information about the value of vaccines from the attending physicians. Engaging in repeated discussions and dedicating more time to patient–doctor interactions could be highly beneficial.

Additionally, inadequate and insufficiently widespread information about vaccines’ efficacy and potential side effects, particularly SARS-CoV-2 vaccines, contributes to patients’ uncertainties and hesitations. Establishing a comprehensive public health vaccination program for individuals with medical conditions is also crucial to ensure convenient and cost-free access, particularly in countries with lower income and financial resources. Lastly, reluctance to embrace vaccination is more common among those with lower socioeconomic status, minority populations [53], and younger age groups [51]. Vigilance and care from healthcare providers and the health system are pivotal in mitigating vaccine disparities, discrepancies, and misinformation propagation. The relationship between disease activity and vaccine efficacy was best studied with the influenza vaccine. In a study of 340 RA patients, increased disease activity did not impair the immune response to the influenza A H1N1 vaccine [54]. While a small study of 24 SLE patients found that those over 50 years of age on prednisone doses greater than 10 mg daily and using azathioprine had a decreased response to the influenza vaccine [55]. This is unlikely reflective of the effect of the medication given that the doses were below the standard definition of immunosuppression per CDC definition [10]. In a study that included 118 juvenile SLE patients and 102 healthy controls [56], patients with significant SLE activity exhibited lower seroconversion rates following influenza A H1N1 vaccination. However, despite the impaired antibody response, the vaccine achieved the standard seroprotection level. 

The various types of SARS-CoV-2 vaccination appear to be less effective in patients with SLE and other AIIRD diseases on immunosuppressive medications, including glucocorticoids, methotrexate, mycophenolate/mycophenolic acid, and rituximab [57]. It is of the utmost importance to emphasize that the benefits of vaccination outweigh the associated risks, even if there is a potential for a decline in efficacy.

Seroconversion rates in immunosuppressed patients compared to immunocompetent individuals remains a subject with limited data. The exclusion of immunosuppressed patients during all phases of SARS-CoV-2 vaccine development has contributed to the scarcity of information in this area [57]. A comprehensive meta-analysis examined the efficacy of SARS-CoV-2 vaccines in immunocompromised patients, encompassing 82 studies [58]. Among these, seven studies specifically focused on patients with AIIRDs. Most of the studies included in the analysis utilized mRNA vaccines. The findings indicated a significant reduction, approximately half, in seroconversion rates among individuals with immune-mediated inflammatory disorders following SARS-CoV-2 vaccination. However, it was observed that seroconversion rates improved notably after the administration of a second dose [58].

While vaccines are typically considered a preventative treatment to strengthen the immune response, emerging studies suggest that vaccines may have the potential to be used as a form of therapy. Vaccine therapy focuses on vaccines as a form of treatment [59]. The concept is based on restoring and retraining the immune system. Although this is still a new area of study in autoimmune diseases, it has been used for other immune system disorders, including allergies [60]. While the idea of decreasing autoimmunity and increasing self-tolerance to self-antigens is promising, there are still concerns regarding selecting the best antigen or autoantigen, with which immune response should be modulated, and the timing of administration [59].

## 7. Safety of Vaccination in AIIRD Patients Receiving Immunosuppressants

### 7.1. Vaccination Adverse Effects 

Adverse reactions can occur after vaccination, despite vaccines being generally considered safe. An adverse event is defined as any unfavorable medical incident that can happen following vaccination, regardless of its potential causal relationship with the vaccine [61]. In addition to the antigen, vaccines may contain various components that can contribute to adverse effects. These components include adjuvants, stabilizers, and preservatives [14]. Vaccine adjuvants present in the human papillomavirus, influenza, and hepatitis B vaccines and other vaccines were among the list of potential triggers of the “Autoimmune/inflammatory syndrome induced by adjuvants (ASIA)” [62]. 

Among 622 patients with AIIRDs who received recombinant zoster vaccination (RZV), adverse effects were reported in 8.7% of cases [63]. Most of these effects were mild such as local reactions, fatigue, arthralgia, or fever, with only four cases of herpes zoster outbreak observed during a median follow-up duration of 36 weeks. In another study involving 403 AIIRD patients, 78.4% of whom were using immunosuppressive medications such as methotrexate, tumor necrosis factor inhibitors, JAK kinase inhibitors, and steroids [64], the rate of adverse effects associated with the Zoster recombinant adjuvanted (ZRA) vaccine was 12.7%. These adverse effects were generally mild, such as fever, pain at the injection site, and skin rash, and three cases of herpes zoster were reported [64].

Live vaccinations include measles, rubella, varicella, mumps, rotavirus, yellow fever, and tuberculosis vaccines. Live vaccines are generally safe; however, infections from some virus strains can occur, albeit mostly mild due to the attenuated nature of the virus [17]. Reassuring results were obtained from a randomized clinical trial on the utilization of live attenuated VZV vaccine among 617 patients with RA and psoriatic arthritis on TNF inhibitors [65]. Over a one-year follow-up period, there were no confirmed varicella infection instances in either the vaccine or placebo group. 

In a 2018 prospective study from Japan, live attenuated vaccines (MMR and varicella) were evaluated in patients with nephrotic syndrome utilizing immunosuppressive therapy [66]. The study included 60 patients, mostly children aged 1–24 years, who were administered a total of 116 vaccinations. The immunosuppressive agents used by these patients included calcineurin inhibitors, antimetabolites such as mycophenolate, or a combination of both, and systemic steroid doses had to be lower than 1 mg/kg/day, and patients on rituximab were excluded. The study showed excellent seroconversion, using virus-specific IgG levels, for measles and rubella and moderate seroconversion for rubella and varicella, which was maintained for 1 year after vaccination. The researchers noted comparable seroconversion rates in another group of immunosuppressed patients, including those with inflammatory bowel disease and rheumatic diseases, who were in a similar age range [67]. 

### 7.2. Safety of Revaccination and Booster Doses

Administering a second booster of the influenza vaccine 3–4 weeks after the initial dose has demonstrated to yield seroprotection levels in immunosuppressed patients with AIIRDs comparable to those observed in healthy individuals [68]. Tdap booster doses in adolescents with juvenile idiopathic arthritis on and off anti-TNF agents were safe and immunogenic [69]. In the Nor-vaC trial, it was observed that administering a third dose of the SARS-CoV-2 vaccine to patients with AIIRDs led to enhanced humoral immune responses that were comparable to those seen in the healthy population who received two doses. The utilized vaccines included BNT162b2, mRNA-1273, and ChAdOx1 nCoV-19, and there was no difference in adverse effects noted between patients and controls following the administration of three and four doses of these vaccines [70,71]. Moreover, an elevated level of protection was observed in immunosuppressed individuals of variable diseases and indications who obtained both full SARS-CoV-2 vaccination and a booster dose with a marked decrease in SARS-CoV-2 infections compared to those who exclusively received the full vaccination regimen [72]. While multiple doses of the SARS-CoV-2 vaccination may improve seroconversion rates among this population, there are challenges in promoting patient vaccination. These challenges include concerns about possible side effects and the financial burden of vaccine costs, especially as government-sponsored vaccination programs are becoming less prevalent.

### 7.3. Risk of Exacerbation of AIIRDs by Vaccination 

A study of 14,928 AIIRD cases, predominantly among women with rheumatoid arthritis, found no increase in primary care visits for rheumatoid arthritis flare-ups after administering inactivated influenza vaccine [73].

A study involving 3554 patients with AIIRDs, with a mean age of 65 years and 68.3% having rheumatoid arthritis, provided data on the risk of AIIRD flare after SARS-CoV-2 vaccination [74]. The study revealed that SARS-CoV-2 vaccination was unrelated to AIIRD flares during the 21 days following vaccination, irrespective of prior COVID-19 infection, the type of AIIRD, and whether mRNA or DNA vaccination technology was utilized.

Among 622 patients with various AIIRDs who received recombinant zoster vaccination (RZV), 16% experienced a disease flare [63]. Specifically, in a subgroup of 403 patients with rheumatoid arthritis (RA), 6.7% experienced a clinical flare following the vaccination [64]. In contrast, the systematic literature review for the 2019 update of EULAR recommendations [33] concluded that live zoster vaccination did not impact the AIIRD activity, even though the small number of patients in these trials limited the finding [33]. 

### 7.4. Vaccination in Pregnant Women with AIIRDs

In a study involving 40 pregnant and 52 breastfeeding patients with AIIRDs [75], pregnant patients reported significantly more adverse events than non-pregnant patients. Still, there was no difference in comparison to pregnant healthy controls. Furthermore, the post-SARS-CoV-2 vaccination disease flare rate was 17.5% and 20% for pregnant and breastfeeding patients, respectively, compared to 18% of the control patients. All patients who experienced flares required glucocorticoids and some needed changes in immunosuppressive treatment. SARS-CoV-2 vaccination during pregnancy in women with AIIRDs is generally safe, with rates of adverse events similar to pregnant healthy controls. The benefits of passive immunization for both mother and fetus outweigh the potential risks [75].

### 7.5. Close Contacts of AIIRD Patients Receiving Immunosuppressants

Both EULAR and the ACR recommendations for the vaccination in adult AIIRD patients highlighted the importance of immunocompetent individuals in households with immunosuppressed patients receiving inactivated and live attenuated vaccines like MMR, rotavirus, varicella, and zoster vaccines per national guidelines but to avoid the oral polio vaccine [29] and smallpox [10]. The varicella vaccine strain can rarely lead to varicella rash, while mumps vaccination can occasionally cause parotid enlargement. There have been reported cases of these conditions in individuals who came into contact with vaccinated individuals [76]. AIIRD patients receiving immunosuppressants should avoid contact with individuals who develop a rash after varicella or zoster vaccination [10,29]. It is recommended that individuals with compromised immune systems avoid contact with the diapers of infants who have recently received the rotavirus vaccine for a minimum of four weeks [10,29].

## 8. Recommendations for the Vaccination of AIIRD Patients Receiving Immunosuppressants 

In addition to the standard vaccinations recommended for the general adult population, such as the seasonal influenza vaccine, hepatitis B vaccine, and SARS-CoV-2 vaccine, international rheumatology societies, such as the American College of Rheumatology [10] and the European Alliance of Associations for Rheumatology [29], recommend vaccinations prioritizing specific vaccinations for patients with AIIRDs who are initiating or undergoing immunosuppressive therapy. This includes vaccinations against *Streptococcus pneumoniae* and herpes zoster, which are more prevalent and pose significant morbidity risks [10,29,77]. Ensuring these patients receive these targeted vaccinations is essential for their comprehensive care. Table 1 summarizes recommended vaccinations for adults with AIIRD receiving or planning to receive immunosuppressive therapy.

### Postexposure Prophylaxis (PEP)

Selected immunosuppressed patients who have been exposed to certain pathogens, including varicella-zoster virus (VZV), influenza virus, COVID-19, hepatitis B virus, measles virus, meningococcus, rabies virus, and tetanus, may require postexposure prophylaxis (PEP). The decision to use PEP is typically based on factors such as the type and timing of the exposure and the level of immunosuppression [81]. It is often recommended to provide PEP for patients who have not been vaccinated against the pathogen they were exposed to and/or for those who are severely immunocompromised [82,83,84].

PEP regimens for specific pathogens may involve antimicrobial prophylaxis, vaccination, and/or passive immunization using generic or pathogen-specific immune globulin [81]. In the case of immunocompromised patients with AIIRD, certain live vaccines recommended in PEP regimens may need to be substituted or supplemented with immunoglobulin administration. For instance, the standard PEP regimen for varicella exposure usually includes vaccination with the live varicella vaccine. However, since most immunosuppressed patients cannot receive live varicella vaccine, VZV immune globulin and/or acyclovir prophylaxis should be administered instead [82].

## 9. Conclusions and Future Directions 

Healthcare providers must prioritize educating AIIRD patients about the increased risks of vaccine-preventable diseases such as COVID-19 and the importance of vaccination. Recommended strategies include timely vaccination during disease quiescence and before initiating immunosuppressants. Enhancing vaccination education and awareness, improving vaccine accessibility, implementing vaccine reminders, addressing vaccine hesitancy, and conducting additional research on vaccine efficacy and safety in this patient population are recommended strategies to increase vaccination rates among patients with AIIRDs. 

In the era of immunosuppression, the future directions of vaccination will likely focus on improving the efficacy and safety of vaccines for individuals with compromised immune systems. In the future, there could be a shift towards developing specialized vaccines tailored to the unique needs of these individuals.

One potential direction is the development of adjuvanted vaccines specifically designed to enhance immune responses in immunosuppressed individuals. By incorporating novel adjuvants or modifying existing ones, researchers can potentially improve the efficacy of vaccines in immunocompromised populations. These adjuvanted vaccines could help boost the immune response and provide better protection against infectious diseases, bridging the gap between the standard vaccine response and the compromised immune system.

Another promising direction is the exploration of alternative vaccine delivery methods. Traditional vaccines often rely on the body’s immune system to produce a strong response. However, in immunosuppressed individuals, this response may be inadequate. Future research may focus on developing vaccines that bypass the compromised immune system by utilizing alternative delivery routes, such as intradermal or mucosal administration. These alternative methods have the potential to activate immune cells directly at the site of administration, potentially leading to a more robust and targeted immune response.

## Figures and Tables

**Table 1:** Recommended vaccinations for adults with autoimmune inflammatory rheumatic diseases (AIIRD) receiving or planning to receive immunosuppressive therapy.

Vaccine Type	Examples	Indications
**Non-live attenuated** (inactivated, subunit, killed, or recombinant)	SARS-CoV-2 vaccine	All patients with AIIRDs who are receiving or planning to receive immunosuppressive therapy [10]
	Pneumococcal PCV15, followed by PPSV23 OR PCV20	All patients with AIIRDs who are receiving or planning to receive immunosuppressive therapy [78]
	Influenza vaccine	All patients should receive a seasonal influenza vaccine annually [79]
	Hepatitis A vaccine	At-risk patients who have not been previously vaccinated [80]
	Hepatitis B vaccine	All patients <60 years old, regardless of risk, and those ≥60 years old with risk factors (e.g., chronic liver disease, injection drug use, household contacts with hepatitis B, and occupational hazard) [80]
	Meningococcal vaccine	At-risk patients who have not been previously vaccinated [29]
	*Haemophilus influenzae vaccine*	
	Human papillomavirus (HPV) vaccine	
	Tetanus, diphtheria, pertussis (Tdap), or tetanus, diphtheria (Td) vaccine	All patients are given per guidelines and schedules as the general adult population [7]
	Recombinant zoster vaccine (RZV)	All patients who are receiving or planning to receive immunosuppressive therapy [10,29]
**Live attenuated**	Measles, mumps, rubella (MMR) vaccine	Patients who have not received prior vaccination against measles and/or do not show proof of immunity to measles (measles IgG seronegative) or those who may have a chance of being exposed to measles should be administered the vaccine before undergoing immunosuppression [77] For AIIRD patients who are at risk of contracting measles infection and are on a low grade of immunosuppression, administering a booster vaccination for the MMR vaccine can be considered [29]
	Varicella vaccine	Patients with uncertain varicella exposure may undergo evaluation of varicella-zoster serostatus to prevent primary varicella infection following the vaccine [29]
	Yellow fever vaccine	AIIRD patients under immunosuppression should avoid yellow fever vaccination due to the risk of infection. Temporarily halting immunosuppressive therapy or checking serology in previously exposed individuals is recommended for AIIRD patients when visiting yellow fever-endemic countries [29]
